# The development of a patient-reported outcome measure for patients with obstructive sleep apnea: the Patient-Reported Apnea Questionnaire (PRAQ)

**DOI:** 10.1186/s41687-017-0021-6

**Published:** 2017-12-15

**Authors:** Inger L. Abma, Maroeska Rovers, Marijke IJff, Bernard Hol, Gert P. Westert, Philip J. van der Wees

**Affiliations:** 1 Radboud University Medical Center, Radboud Institute of Health Sciences, IQ healthcare, PO box 9101, huispost 114, 6500 HB Nijmegen, The Netherlands; 2 Radboud University Medical Center, Radboud Institute of Health Sciences, Departments for Health Evidence and Operating Rooms, Nijmegen, The Netherlands; 3 ApneuVereniging, Doorn, The Netherlands; 4 Albert Schweitzer Ziekenhuis, Dordrecht, The Netherlands

**Keywords:** Obstructive sleep apnea, Quality of life, Instrument development, Patient-reported outcome measure

## Abstract

**Background:**

Obstructive sleep apnea (OSA) is a chronic condition that can have a wide range of consequences for a patient’s health-related quality of life. Monitoring aspects of quality of life in clinical practice has the potential to improve the patient-centeredness of care for patients with OSA. The aim of this article is to describe the development of the Patient-Reported Apnea Questionnaire (PRAQ), a patient-reported outcome measure (PROM) that is designed for use in clinical practice on an individual patient level, as well as subsequent outcome measurement on an aggregate level.

**Methods:**

We used the items of available PROMs for OSA to create a new PROM with focus on its applicability in clinical practice. We used a tailored development process to come to a selection of domains and items. Patients and healthcare professionals were intensively involved in the development of the PRAQ via membership of the development team, online surveys and focus groups, as well as two rounds of cognitive validation.

**Results:**

This first version of the PRAQ consists of 43 items and 10 preliminary domains, and covers the aspects of quality of life that healthcare professionals and patients wish to discuss in clinical practice. Patients indicate that PRAQ is comprehensive and that its length is acceptable. Comprehensive patient involvement has ensured good content validity for the PRAQ.

**Conclusions:**

This article shows how a PROM can be developed with a specific focus on its applicability in clinical practice.

## Background

OSA is a highly prevalent, chronic condition in which temporary obstructions of the upper airway cause breathing stops while asleep [1]. Arousal of the brain in patients with OSA results in continuation of breathing, which often goes unnoticed by the patient but can happen up to hundreds of times per night. This causes fragmented sleep and can result in severe sleepiness, fatigue and impaired mood during the day, which in turn can affect a patient’s relationships, psychological wellbeing, cognitive functioning, and participation in work and other activities [2–7]. Furthermore, OSA has been recognized as an independent risk factor for hypertension, heart failure, and diabetes [8–10]. The general population prevalence of OSA has been reported to be 13 to 33% in men and 6 to 19% in women [11], but in practice OSA goes undiagnosed in many patients [3, 12, 13].

The wide range of consequences and the chronic nature of OSA make focus on health-related quality of life (HRQoL) during the care process highly relevant. HRQoL is quality of life relative to one’s disease status [14] and has been captured in several models [14–16]. Patient-reported outcome measures (PROMs) are questionnaires which are filled out by patients with the aim of measuring symptoms, daily functioning or Health-Related Quality of Life (HRQol). Most of the currently existing PROMs were developed for research purposes, to measure the impact of interventions on perceived health in clinical trials [17]. In recent years, the use of these existing PROMs has also expanded to areas closer to daily clinical practice [18–20]. There, individual PROM scores are used for the detection of problems with HRQoL, monitoring a patient’s response to treatment, and to improve patient-centeredness of care by directing more attention to a patient’s quality of life during consultations with healthcare professionals [21]. Furthermore, PROMs can be used as outcome measures to assess the quality of treatments or providers [22]. The integrated use of PROMs for these different purposes, which includes PROM measurements at both intake and during follow-up, is expected to stimulate meaningful use in clinical practice and quality improvement [23].

A recently published systematic review [24] identified three available PROMs that were developed specifically for and with patients with OSA, and which aim to measure quality of life. However, the focus during their development was only on outcome measurement. Furthermore, because of either practical reasons (the PROM has to be administered by an interviewer) or content reasons (omission of important aspects of quality of life, and unclear phrasing of some of the items) these PROMs did not seem suitable for use in clinical practice. Therefore, we decided to develop a new PROM that covers the topics that patients and clinicians find relevant to discuss with regard to apnea-related quality of life, and which is also suitable for outcome measurement.

The aim of this article is to describe the development of a new PROM, the Patient-Reported Apnea Questionnaire (PRAQ), that measures the different aspects of OSA-related quality of life. This PROM can help focus clinical practice on the HRQoL of an individual patient, and can subsequently be used as an outcome measure for quality assessment.

## Methods

In developing the new PROM we used a set of steps that would ensure thorough patient and clinician involvement. These steps follow the general PROM development process as described in the literature [25–27]: item generation based on patient interviews or focus groups, selecting the items, developing scales and scoring method, and pilot testing the items (cognitive validation). Our approach to item generation phase was different from that described in the literature, as we pooled the items of existing PROMs rather than generating ourselves. However, the item generation of these PROMs was based on patient input [28–30]. Additionally, during the item selection process of the PRAQ, we also gathered information specifically on the suitability of the domains and items for a PROM which will be used in clinical practice. We undertook the following steps:

1. forming a working group with different stakeholders;
2. creating a preliminary pool of items from existing PROMs and sorting these items into preliminary domains based on the topics of the items;
3. using a patient survey and healthcare professional survey to gather input for domain and item selection;
4. selecting domains and items with the working group;
5. discuss and adapt this selection in patient focus groups;
6. performing two phases of cognitive validation.

Each of these steps is explained in further detail in the following paragraphs. The definite sorting of the PRAQ items into domains with the help of psychometric methods will be conducted after a follow-up study and is outside the scope of this article.

### Forming a working group

A working group was formed consisting of two researchers (IA and PW), a board member (MI) of the patient organization for OSA in The Netherlands (ApneuVereniging), and a pulmonologist specialized in OSA (BH), based at the Albert Schweitzer Hospital in Dordrecht, The Netherlands. The working group made the necessary decisions for the PROM development throughout the development process, based on the input from patients and healthcare professionals whenever possible.

### Creating preliminary pool of items

Three available PROMs which were previously developed for patients with OSA used patient input for the creation and/or selection of items [24]: Sleep Apnea Quality of Life Index (SAQLI) [28], Quebec Sleep Questionnaire (QSQ) [29, 31], and the Maugeri Obstructive Sleep Apnea Syndrom (MOSAS) questionnaire [30]. In the opinion of the working group, the QSQ and MOSAS questionnaire appear to miss some important topics, e.g. items about emotions or symptoms, respectively. Furthermore, the phrasing of some items was deemed suboptimal. The SAQLI appears unfeasible for use in clinical practice because it is interviewer-administered, but it does cover a very broad range of topics identified by patients in its development phase. Therefore, the working group decided to create a pool of items consisting of the items of these three PROMS, and use these items to create a new PROM which covers all relevant issues and which also suits our different purposes. We decided to use a 7-point Likert scale similar to that used in the QSQ and SAQLI. Seven response options have shown to be more reliable than 5 response options, possibly because raters do not like to choose the two most extreme response options of a scale [27]. After discussion in the working group we also decided to keep the 4-week recall period, because patients with OSA generally struggle with symptoms over longer periods of time and our patient representative indicated that shortening this period may feel too restrictive for patients. Four weeks is the maximum recall period that is recommended for this type of questionnaire [32], is suited to the effects of therapy on a chronic illness [33] and is also used in well-known PROMs such as the SF-36 [34].

The three PROMs were each translated into Dutch by two translators who are native Dutch speakers. The working group selected the translation considered optimal for each item. We did not perform a backwards translation because we did not aim to adhere to the exact phrasing of the items: we only wanted to keep the topics the same. The working group and particularly the patient representative paid specific attention to whether the translated items and topics made sense the context of measuring quality of life for patients with OSA, to ascertain that the translators had not made misinterpretations. Furthermore, the working group made sure that all items were suitable for patients that were suspected of having OSA, as well as patients already diagnosed with or treated for OSA, and that items were suitable to potentially measure change over time.

All items of these three PROMs together formed our pool of items. When items from different PROMs were highly similar in both phrasing and topic, only one of the items was kept in our item pool. The working group then grouped the items into preliminary domains according to their topic, keeping in mind the conceptual model of health-related quality of life developed by Wilson and Cleary [16], separating items on symptoms and functional status.

### Gathering information for item selection: patient and healthcare professional survey

An online patient survey was distributed to gain input for item and domain selection, covering how important the different items are for patients with OSA (on a scale of 1 to 9); whether any items or domains are missing; and which topics patients would like to discuss with an OSA physician or nurse. Patients were also asked to comment on the phrasing of the items and to indicate if they found any items hard to understand or confusing. The survey was sent out to patients with OSA and partners of patients with OSA who are volunteers of the Dutch patient organization for OSA. These volunteers have encountered many patients with OSA in their volunteer work, and were asked to base their importance ratings for the individual items on their expertise based on this broad experience.

An online survey for healthcare professionals was set up to gain the following information per domain: to what extent respondents would want to know if their patients had these kind of problems; to what extent they thought treatment for OSA would reduce these problems; and to what extent they considered themselves at least partially responsible for helping to solve these problems for their patients – which includes the option of referring patients to another healthcare professional, such as a psychologist. Furthermore, the respondents were asked if they thought any domains were missing.

### Preliminary domain and item selection

The working group selected the domains and subsequently the items of the PRAQ based on the surveys. We considered a domain relevant if more than 50% of both patients and healthcare professionals answered positively on the questions regarding whether a patient would want to discuss this domain with their healthcare provider, or the other way around, i.e. whether a healthcare provider was interested to learn more on this domain topic form the patient (a score of 7, 8 or 9 was considered positive). If this criterion was not met, the domain was up for discussion in the working group and patient focus groups.

As a next step, the working group selected the items within the selected domains. We excluded all items considered important by less than 50% of patients, items considered important by 50–70% of patients were up for discussion in the working group. Additionally, items were adjusted and potentially included if there were specific comments explaining why the score of item was low, such as issues around comprehensibility of the item. Patient comments were also used to identify items which were considered highly similar, and we discarded those with the lowest importance score.

### Discussing the preliminary item selection: patient focus groups

After the preliminary selection of items by the working group, two patient focus groups (*n* = 9 and *n* = 5 participants, with at least two women in each) were held to discuss the results and the choices of the working group, and to reaffirm the relevance of the items for patients with OSA. Participants were volunteers of the OSA patient organization who had completed the survey.

### Cognitive validation

Two phases of cognitive validation [35, 36] were carried out, each involving six patients with OSA or suspected OSA attending a consultation at a sleep centre, and if present, their partners. Ages of the patients ranged from 42 to 74, and highest education level ranged from primary school to undergraduate college. Half of the included patients were women. For one of the patients, Turkish was their first language.

The aim of the cognitive validation was to check whether all selected items were understood by patients as intended, and whether the answering options were complete and made sense. All patients were asked to think aloud while completing the PROM, and were asked additional questions (probing) about their interpretation of the items. Items that were unclear were either removed or adjusted. Subsequently, a second phase of cognitive validation was carried out with the adjusted PROM.

## Results

**Fig. 1:**
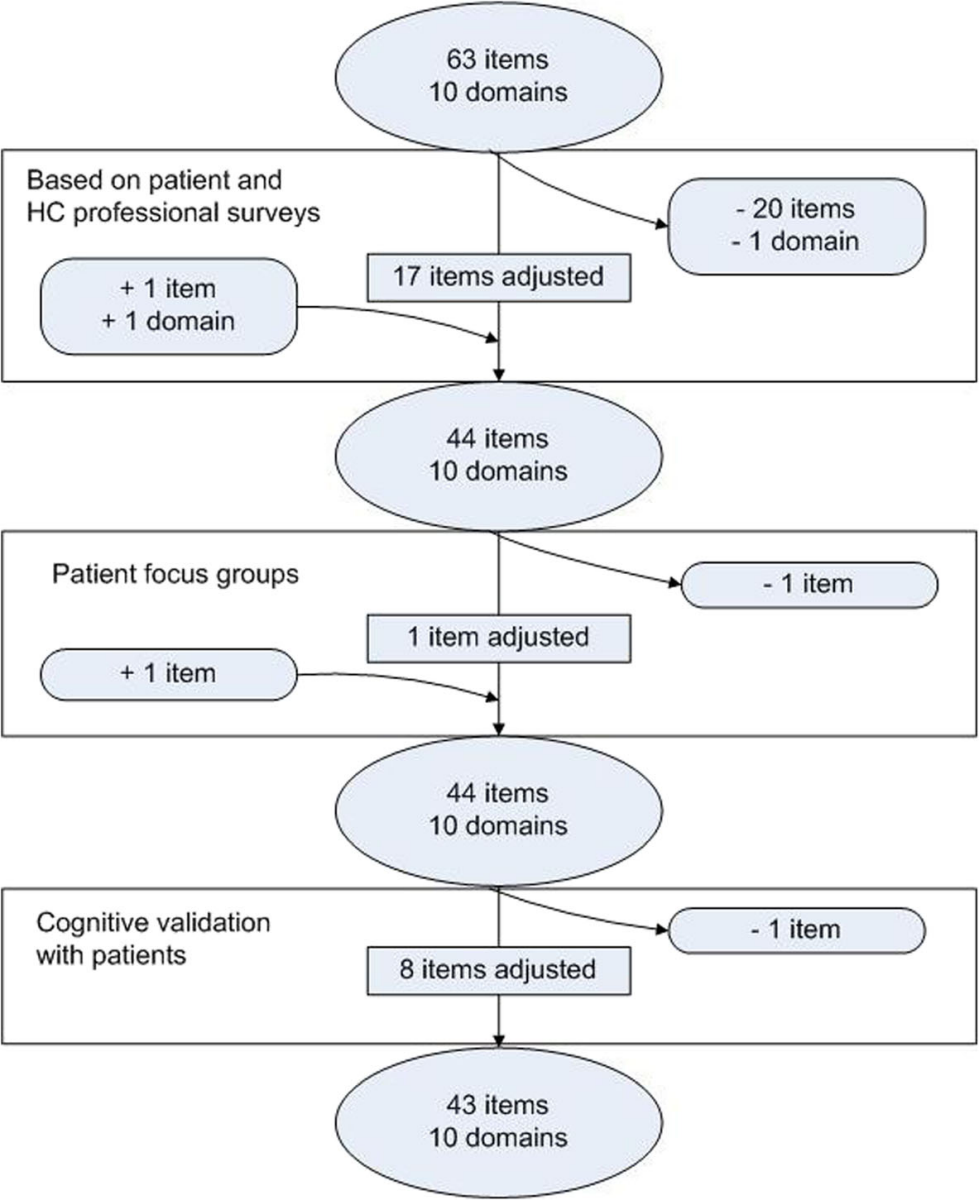
Flow chart of the PRAQ development process

The development of the PRAQ is summarized in the flow chart in Fig. 1.

### Creating preliminary pool of items

Our preliminary pool of items consisted of 63 items, which the working group sorted into 10 preliminary domains: symptoms at night, sleepiness, tiredness, memory & concentration, unsafe situations, concerns about health, daily functioning, direct effect of apnea on others (e.g. bothering others due to snoring), social interactions (with sexuality as a subtopic), and emotions.

### Patient and healthcare professional surveys

The patient survey was sent out to 85 volunteers of the Dutch OSA patient organization, of which 35 people completed the survey (41%). The characteristics of the respondents can be found in Table 1.

**Table 1:** Patient survey respondent characteristics

Total nr of respondents	35
Current patients with OSA	30
Former patients with OSA	3
Partners	2
Gender	29% female
Median age category	60–69
Patients only (*n* = 30)	
Treatment by CPAP/BPAP	100%
CPAP with additional MRA or operation	13%

*BPAP* bilevel positive airway pressure, *CPAP* continuous positive airway pressure, *MRA* mandibular repositioning appliance, *OSA* obstructive sleep apnea

Most of the individual items were considered important (7 or higher on a scale of 1–9) by a majority of respondents (70–90%). The items in the “social interactions” domain had generally lower scores than the items in other domains, with a range of 29–71% of patients regarding them as important. Within this domain, the question about sexuality was considered important by the most respondents.

There was a general desire to be able to discuss the ten domains in a consultation with an OSA healthcare professional. Looking at the percentage of patients that scored their desire to discuss a certain domain with at least 7, the highest scores were for the domains “daily functioning” (88%) and “symptoms at night” (87%). The lowest scores were for direct effect of apnea on others (65%), social interactions (60%) and sexuality (55%).

The healthcare professional survey was sent out to 55 OSA professionals of whom 30 completed the survey (55%). The characteristics of the respondents can be found in Table 2.

**Table 2:** Healthcare professional survey respondent characteristics

Respondents (*n*)	30
Employed at n sleep centers	26
Median age category	50–59
Gender	53% female
Physician (*n*)	16
Pulmonologist (*n*)	10
Otolaryngologist (*n*)	2
Neurologist (*n*)	4
OSA nurse (*n*)	12
OSA nurse practitioner (*n*)	2

*OSA* obstructive sleep apnea

For each of the domains, the majority of healthcare professionals indicated that they would want to know if their patients had problems with it (82–100%). Most of them also felt at least partially responsible for helping to solve these problems, either by treating the patients themselves or referring the patient to another healthcare professional (72–100%). Most healthcare professionals felt that treating their patients’ OSA would improve complaints about sleepiness, symptoms at night, and the direct effect of apnea on others (89–96%). For the other domains, opinions were more diverse. Problems with sexuality were considered “likely to improve” by the fewest survey respondents (46%).

### Preliminary selection of topics and items

A majority of both healthcare professionals and patients wanted to be able to discuss each of the ten preliminary domains, so we considered all domains relevant for the PRAQ.

We decided to keep the item asking about anxiety in the domain “emotions” despite its relatively low importance scores on the patient survey (60% of patients considered this an important item to add, versus over 70% for other items), because anxiety is more common in female patients with OSA [37, 38] and women were slightly underrepresented in the sample.

After item selection, only one item remained as part of the domain “direct effect of OSA on others”, so we decided to move this item to “social interactions”.

Patients indicated that additional items about sleep problems should be part of the PRAQ, which was supported by the results of the healthcare professionals. Therefore, we added the domain “quality of sleep”, covering the suggested sleep problems. This resulted in a total of ten preliminary domains and 44 items.

### Patient focus groups

During the patient focus groups, the preliminary selection of domains and items was discussed. One item was adapted, and one item was added to the domain “emotions” about experiencing sudden, intense emotions. One other item for this domain (“how often did you feel you were unreasonable?”) was removed after discussion in the group. The participants felt that a patient was unlikely to admit to being unreasonable, and that this type of emotion would be sufficiently covered by the items about feeling irritable and losing one’s temper.

The number of answering categories was discussed, as several patients preferred to have ten answering categories rather than the proposed seven options, because this be similar to the scores of the Dutch version of a report card in school and thus would be more intuitively understandable. However, there was no consensus about this in the focus groups. We decided with the working group to maintain the 7-point Likert scale, for two reasons: scoring the PRAQ like a report card might give patients the idea that they are being judged on how well they are “performing”, which is not desirable; and as stated before, in the literature seven answering categories are often thought to be optimal [27].

Patients also commented on the recall period of the items: recalling symptoms of the past four weeks was generally seen as too short a time period. Newly diagnosed patients have often been experiencing symptoms for years, and choosing a long recall period, e.g. six months, would be more relevant for this particular group. However, follow-up appointments for CPAP users can be as early as four to six weeks after initiating treatment. Since we would like our PROM to be a useful addition to follow-up appointments as well as the intake appointment, using a recall period of more than four weeks is not desirable. To address the wishes of the patients, we therefore added an open text field at the end of each domain in which patients get the opportunity to describe past symptoms.

We also discussed the acceptable number of items for the new PROM. Patients of both focus groups felt that all remaining items were relevant and important, and that the length of the PRAQ was acceptable. The exception was the domain “sleepiness” (containing eight items), which patients said could likely be further reduced without information loss. As there was no patient preference for which items should remain in the selection, we will perform the final selection of items for this domain with psychometric methods after a pilot study has taken place.

After the focus groups, there were 44 items left for the PRAQ.

### Cognitive validation

Twenty-one patients were interviewed, aged between 42 and 74 years and with different education levels. There were several items in the PROM that were confusing to all or most of the interviewed patients, or that they understood in a way that was not intended, which were subsequently adjusted or removed. One example of a misunderstood item was “Were you concerned about your safety or that of others in traffic or while operating machinery?”. Several patients indicated concern about their safety in traffic because they thought *other people* were often bad drivers. We adjusted this question to include “due to your sleepiness”, to shift the focus of this item to the patient’s own potential problems due to OSA. During the second phase of the cognitive interviews, the meaning of the newly adjusted items as well as the other items in the current item selection was clear to the patients.

### Final result

Based on the development process described in this paper, the current PRAQ comprises ten (preliminary) domains and 43 items, and takes approximately 15 min to fill out. The official English translation of this preliminary PRAQ can be found in Table 3. In a next stage of development, which involves a validation study assessing reliability, validity and responsiveness, final item selection and domain construction will take place.

**Table 3:** English translation of preliminary PRAQ^a^

Symptoms at night	
During the past 4 weeks, did you have a problem with: 1. Snoring loudly? 2. Waking up frequently to urinate? 3. Waking up at night with the feeling that you are choking? 4. A feeling that you are sleeping restlessly? 5. Having a dry or painful mouth when you wake up? 6. Waking up in the morning with a headache?	
Sleepiness	
During the past 4 weeks, did you have a problem with: 7. Fighting to stay awake during the day? 8. Suddenly falling asleep? 9. Difficulty staying awake during a conversation? 10. Difficulty staying awake while watching something? (concert, movie, television) 11. Falling asleep at inappropriate times or places? 12. Difficulty staying awake while reading? 13. Fighting to stay awake when you are driving? 14. Did you feel like you needed to take a nap in the afternoon?	
Tiredness	
During the past 4 weeks, did you have a problem with: 15. Feeling very tired? 16. Lacking energy? 17. Still feeling tired when you wake up in the morning?	
Daily activities	
During the past 4 weeks: 18. How difficult was it for you to do your most important daily activity? (such as your job, studying, caring for the children, housework) 19. How often did you use all your energy to accomplish only your most important daily activity? (such as your job, studying, caring for the children, housework) 20. Did you feel you have a decreased performance with regard to your most important daily activity? (such as your job, studying, caring for the children, housework) 21. How much difficulty did you have finding energy for your hobbies? 22. How difficult was it for you to get your chores done?	
Unsafe situations	
During the past 4 weeks: 23. Did you have problems while driving a car due to sleepiness? 24. Were you concerned about your safety or that of others due to your sleepiness? (for example in traffic, or when operating machinery)	
Memory and concentration	
During the past 4 weeks: 25. Were you sometimes forgetful? 26. Did you sometimes have difficulty concentrating?	
Quality of sleep	
During the past 4 weeks, did you have a problem with: 27. Falling asleep when you go to bed at night? 28. Getting back to sleep after you woke up at night?	
Emotions	
During the past 4 weeks: 29. How often did you feel depressed or hopeless? 30. How often did you feel anxious? 31. How often did you lose your temper? 32. How often did you feel that you could not cope with everyday life? 33. How often did you feel irritated? 34. How often did you have a strong emotional reaction to everyday events?	
Social the past 4 weeks: 35. Did you sometimes feel upset because others were disturbed by your snoring? 36. Was it a problem for you that you sometimes had no energy or no desire to do things with your family or your friends? 37. Did you feel guilty towards your family or friends? 38. Did you feel upset because you argued frequently? 39. Did you sometimes experience problems in the relationship with your partner? 40. Did you feel upset because you could (maybe) not sleep in the same room as your partner? 41. Did you sometimes think up excuses because you were tired or sleepy? 42. Did you have a problem with unsatisfying and/or too little sexual activity? (by yourself or with another)	
Health concerns	
43. Were you concerned about other conditions that may be related to sleep apnea? (such as diabetes, high blood pressure, cardiovascular disease, being overweight)	

^a^The PRAQ was translated into English by an official translator who is a native English speaker, and by IA. The translator, IA and PW together reached consensus on the translation of each item. The English PRAQ was translated back into Dutch by another official native Dutch translator, and IA and PW used input from this translator to adapt the English version where needed

## Discussion

In this article we describe the development of a quality of life PROM for patients with OSA, the Patient-Reported Apnea Questionnaire (PRAQ). The PRAQ was developed with the goal of serving as a useful addition to daily clinical practice at an individual patient level, to help focus more attention on quality of life, and subsequently as an outcome measure for quality assessment. We developed the PRAQ by using the pooled items of existing PROMs for patients with OSA and subsequently adapted and selected items with the input of physicians and patients. This resulted in a preliminary PRAQ with 10 domains and 43 items.

Item selection for the PRAQ is not yet entirely complete: within the domain of “sleepiness”, patients felt that the number of items could be reduced. However, they had no opinion on which of the eight items should be removed, because they were all relevant. Psychometric methods will be used to reduce the number of items in this domain using the data of a pilot study on this preliminary version of the PRAQ.

Next to being used on an individual patient level in clinical practice, our aim for the PRAQ was to be able to use its outcomes for quality assessment at an aggregate level. One important PROM measurement quality specifically for quality assessment is that a PROM must be *responsive*, i.e. that it is able to measure changes in a patient’s condition over time. We took this into account in the development of the PRAQ by asking healthcare professionals whether they expected that different aspects of quality of life, as covered by the preliminary domains, would improve after treatment for OSA. The actual responsiveness of the PRAQ will be assessed in a validation study.

When developing a PROM that can be used both on an individual patient level in daily clinical practice and as an outcome measure for quality, it is important to find a balance between the wishes of patients and the requirements for creating a feasible outcome measure. For example, the patients wished to communicate symptoms from as far as six months ago, which is not a feasible recall period for a a quality of life PROM [27, 32]. We believe that the solution the working group devised together with the patients – offering patients an open text field for each domain, which can be used in clinical practice, if not for the scoring of the PRAQ – is a reasonable compromise in this case.

Patient input is very important during the PROM development process [39, 40]. For our patient input, we made use of the knowledge and experience of volunteers of the OSA patient organization in The Netherlands. Such volunteers are a relatively engaged population, and might therefore differ slightly from regular patients with OSA, but we do believe that they were able to give an accurate representation of what is important to this patient group. Furthermore, because we also used the input of healthcare professional and available literature, we do not believe that any important domains are missing.

To develop the PRAQ we used the items of the SAQLI, QSQ, and the MOSAS questionnaire [28, 30, 31]. Even though the PRAQ contains many items that are similar to the items of these PROMs, it also differs from them substantially. Compared to the SAQLI, the PRAQ is shorter and easier to understand, allowing patients to complete the PROM without an interviewer. The PRAQ is more elaborate than the QSQ in its emotions and social functioning domains. Furthermore, quite a few items of the QSQ and MOSAS questionnaire were seen as unclear or less relevant by the patients with OSA in this study and were therefore not added to the PRAQ. Furthermore, the preliminary PRAQ has its items grouped into more domains than the other PROMs, because we split up the symptoms that patients can experience during the day in more separate domains than the other PROMs. We chose this approach because we wanted to create domains of which the scores are more easily interpretable by healthcare professionals. A future factor analysis, as part of the validation of the PRAQ, will have to show whether the way items are currently grouped into domains makes sense psychometrically. Based on the validation study, the items of the PRAQ will then be sorted into their final domains.

The method used for developing this PROM, which includes forming an item pool out of the individual items of existing PROMs, can be employed by others as is, or can be adjusted to fit the needs of a specific situation. The thoroughness of prior research and the number of PROMs and items available will have to be taken into account when deciding on the exact approach of the development process. If it is suspected that currently available PROMs do not cover all aspects which are important to the patient population, additional research may be warranted to expand the item pool, for example in the form of patient interviews.

### Future research

Our next step will be to perform a pilot study to finalize item selection and the formation of domains for the PRAQ. We subsequently study the measurement properties of the PRAQ, to estimate its suitability for measuring outcomes for individual patients and for quality assessment at an aggregate level. We will also develop a digital tool which summarizes the results of the PRAQ in a patient-friendly manner for use during consultations.

## Conclusions

This article shows how a PROM can be developed with a specific focus on its applicability in clinical practice. This study resulted in the preliminary version of a PROM for patients with sleep apnea: the Patient-Reported Apnea Questionnaire (PRAQ), containing 10 domains and 43 items.
